# Cost-effectiveness of screening and treating alcohol use and depression among people living with HIV in Zimbabwe: a mathematical modeling study

**DOI:** 10.1186/s12916-024-03674-8

**Published:** 2024-10-21

**Authors:** Jasmine I-Shin Su, Yao-rui Yeo, Mellesia Jeetoo, Neo K. Morojele, Joel M. Francis, Sheela Shenoi, R. Scott Braithwaite

**Affiliations:** 1 Department of Population Health, New York University Grossman School of Medicine, 227 E. 30th St, New York, NY 10016 USA; 2 Department of Psychology, University of Johannesburg, Cnr Kingsway and University Roads, Auckland Park 2092, Johannesburg, South Africa; 3 Department of Family Medicine and Primary Care, School of Clinical Medicine, Faculty of Health Sciences, University of the Witwatersrand, 29 Princess of Wales Terrace, Parktown, 2193, Johannesburg, South Africa; 4 Yale AIDS Program, Yale School of Medicine, 135 College Street, Suite 323, New Haven, CT 06510 USA

**Keywords:** HIV infections, Cost-effectiveness analysis, Alcohol use disorder, Depression, Zimbabwe, Sub-Saharan Africa, Mathematical modeling

## Abstract

**Background:**

Alcohol use disorder (AUD) and major depressive disorder (MDD) drive HIV transmission in many sub-Saharan African settings. The impact of screening and treating AUD and MDD on HIV outcomes is unknown. We aimed to identify the cost-effectiveness of AUD and MDD interventions in Zimbabwe, and their potential contribution to reaching Zimbabwe’s Ending the HIV Epidemic 2030 goal.

**Methods:**

Using a validated HIV compartmental transmission model in Zimbabwe, we compared four policy scenarios: prevention as usual (baseline); implement AUD screening (using AUDIT) and treatment (motivational interviewing and cognitive-behavioral therapy); implement MDD screening (using PHQ-9) and treatment (cognitive-behavioral therapy); and implement screening and treatment for both. Outcomes were HIV incidence projections, infections averted through 2030, quality-adjusted life-years gained, cost per infection averted, and cost per QALY gained. Analyses considered “spillover,” when treatment for AUD also results in an improvement in MDD and the converse. Sensitivity analyses identified cost reductions necessary for AUD and MDD interventions to be as cost-effective as other HIV interventions, particularly the scale-up of long-acting PrEP.

**Results:**

AUD and MDD combined will be responsible for 21.1% of new HIV infections in Zimbabwe by 2030. *Without considering spillover*, compared to the baseline, MDD intervention can reduce new infections by 5.4% at $2039/infection averted and $3186/QALY. AUD intervention can reduce new infections by 5.8%, but at $2,968/infection averted and $4753/QALY, compared to baseline. Both MDD and AUD interventions can reduce new infections by 11.1% at $2810/infection averted and $4229/QALY, compared to baseline. *Considering spillover*, compared to the baseline, MDD intervention can reduce new infections by 6.4% at $1714/infection averted and $2630/QALY. AUD intervention can reduce new infections by 7.4%, but at $2299/infection averted and $3560/QALY compared to baseline. Both MDD and AUD interventions can reduce new infections by 11.9% at $2247/infection averted and $3382/QALY compared to baseline. For MDD intervention to match the cost-effectiveness of scaling long-acting PrEP, the cost of MDD intervention would need to be reduced from $16.64 to $12.88 per person.

**Conclusions:**

Implementing AUD and MDD interventions can play an important role in HIV reduction in Zimbabwe, particularly if intervention cost can be decreased while preserving effectiveness.

**Supplementary Information:**

The online version contains supplementary material available at 10.1186/s12916-024-03674-8.

## Background

It is a national priority for Zimbabwe to reach its Ending the HIV Epidemic (EHE) goal of reducing HIV incidence by 90% between 2010 and 2030. The constellation of alcohol, substance, and mood-related (CASM) conditions augment a person’s risk of contracting HIV [1–5]. CASM conditions include alcohol, tobacco, and other substance use disorders, as well as depressive disorders, anxiety disorders, and chronic pain, which often co-occur with each other. However, the current HIV intervention portfolio in Zimbabwe does not include screening and treatment for CASM conditions, and people with HIV (PWH) are not screened regularly for these conditions in HIV clinics [6, 7].

Those with CASM conditions are more likely to engage in risky sexual behaviors, such as condomless sex, thereby increasing the risk of HIV transmission [1]. Individuals with alcohol use disorder (AUD), for instance, are 70% less likely to be tested for HIV in South Africa, compared to people without AUD [2]. Among those with a positive HIV diagnosis, individuals with AUD are 65% less likely to be linked to care [8]. CASM conditions such as depression, anxiety, and AUD also reduce antiretroviral therapy (ART) adherence [3, 4]. A microsimulation study of PWH in the US suggested that CASM screening and treatment can reduce HIV/AIDS-related deaths and raise life expectancy [5]. Screening and treatment for CASM conditions thus have the potential to impact a wide array of HIV outcomes.

The interdependence of CASM conditions further increases the potential impact that screening and treating CASM can have on the HIV epidemic in Zimbabwe. Having one CASM condition increases the probability of having another CASM condition (“overlap”) [5]. Remission in one CASM condition also increases the probability of remission in another condition (“spillover”) [9]. Overlap and spillover augment the potential benefit of CASM screening and treatment, and research is needed to evaluate their impact in Zimbabwe’s context. In this study, we explore the potential effects of adding screening and treatment of alcohol use disorder (AUD) and major depressive disorder (MDD) to Zimbabwe’s current HIV intervention portfolio, using a validated simulation model that projects HIV incidence in the Zimbabwean population over a 10-year time horizon.

## Methods

We adapted a previously published HIV compartmental transmission model for the Zimbabwe setting, calibrating it to predict HIV prevalence and incidence since the approximate onset of HIV (1990; Additional file: Figs. S4–S7) [5]. The model simulated the effects of CASM interventions on HIV diagnosis, treatment, and viral load suppression. We incorporated the screening and treatment for AUD and the screening and treatment for MDD to represent CASM interventions. Prevalence of other substance use disorders, such as tobacco use and opioid use disorders, is anecdotally low in Zimbabwe, so our evaluation of CASM focused on AUD and MDD.

We modeled the Zimbabwean population from 2020 through 2030 and beyond. Compartments in the model included sex (male/ female), age (< 15/15–24/25–34/35–64/ ≥ 65), risk types for men (men who have sex with men [MSM]/non-MSM), risk levels (low risk/medium risk/high risk), AUD status (have AUD/no AUD), MDD status (have MDD/no MDD), and genotypic resistance (wildtype HIV/ resistant HIV). High-risk groups were defined as people with large numbers of concurrent sexual partners (e.g., commercial sex workers), medium-risk groups were others who were in non-monogamous relationships in the past year, and low-risk groups were sexually active people in monogamous relationships. Those who are not sexually active are considered as no risk. Individuals with a score of 8 or more on the Alcohol Use Disorders Identification Test (AUDIT) were considered as having AUD, and those with an AUDIT score < 8 were considered as not having AUD. Individuals with a score of 10 or more on the Patient Health Questionnaire (PHQ-9) were considered as having MDD, and those with PHQ − 9 score < 10 were considered as not having MDD. Definitions for AUD and MDD followed the literature [10, 11]. We assumed that no formal diagnostic assessment followed screening.

### Policy comparisons

Starting from 2020, four policy scenarios were considered through 2030: implement neither AUD nor MDD interventions (baseline), implement AUD intervention only, implement MDD intervention only, and implement both AUD and MDD interventions. Model outcomes were evaluated at year 2030 because Zimbabwe’s EHE goal aims to reduce HIV incidence by 90% from 2010 to 2030.

**Fig. 1:**
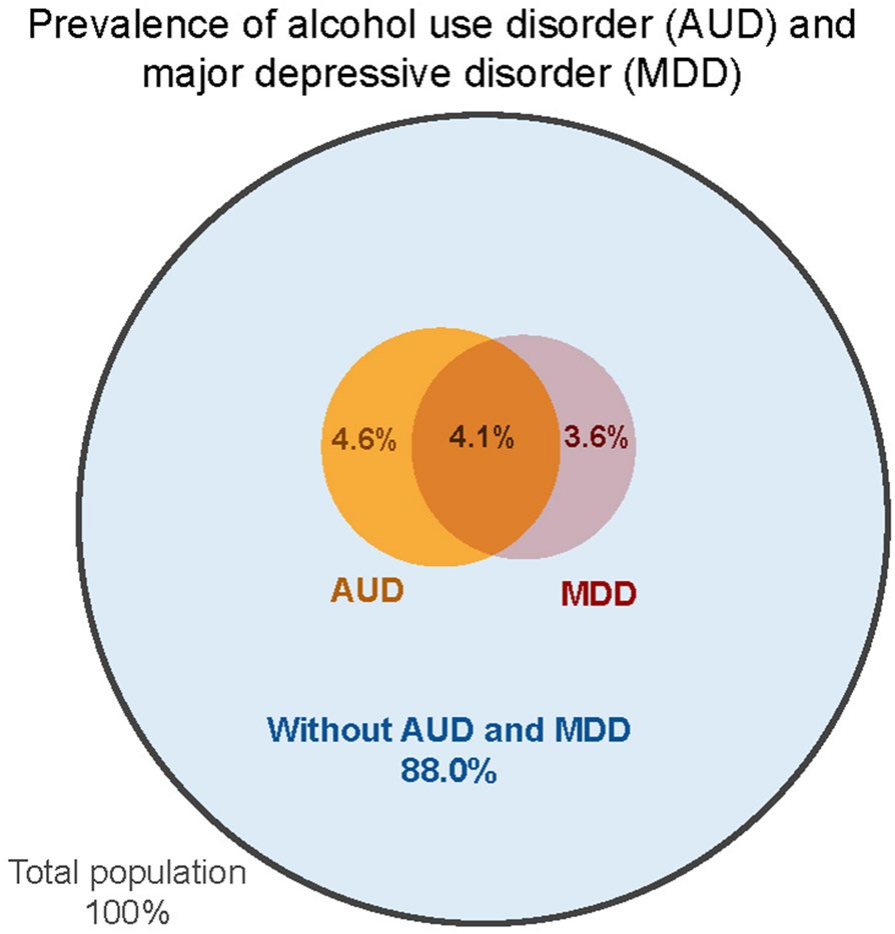
Prevalence of alcohol use disorder and major depressive disorder in the model

**Fig. 2:**
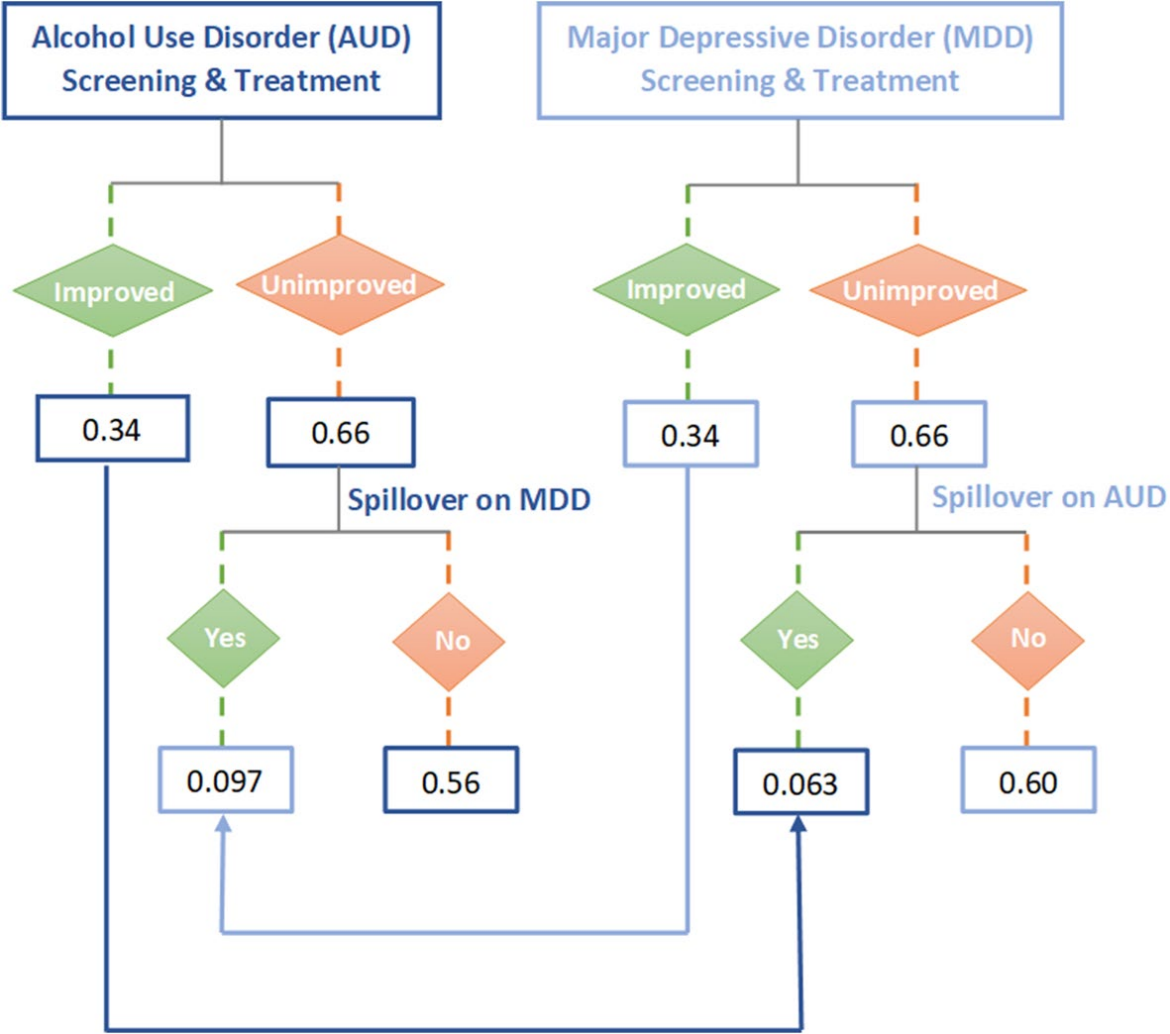
Effectiveness inputs of policy scenarios

Based on published literature, we determined that at baseline, 8.7% of the Zimbabwean population have AUD and 7.7% have MDD (Table 1; Fig. 1) [7, 12–20]. Among those with AUD, 47.5% also have MDD, and 53.2% of those with MDD also have AUD (Table 1) [5, 21–24]. Effectiveness of CASM treatment was obtained from published literature (Table 1; Fig. 2), and so were the sensitivity and specificity of CASM screening instruments (Table 1) [21, 23].

**Table 1:** Key model inputs

Parameter	Value		Reference
***Total population***			
Age			
0–14	38.3%		[12]
15–64	57.2%		Calculated; [12, 13]
65 +	4.5%		[12]
HIV risk behaviors	**Female**	**Male**	
Abstinent	36.3%	29.1%	[25–27]
Low risk (monogamous)	52.7%	44.0%	[26]
Medium risk (non-monogamous)	9.8%	23.2%	[25]
High risk (sex workers or clients of sex workers)	1.2%	3.7%	[27]
***Alcohol use disorder (AUD) and major depressive disorder (MDD)***			
Prevalence			
AUD, no MDD	4.6%		Calculated; [7, 12–18]
MDD, no AUD	3.6%		Calculated; [7, 12–17, 19]
Both AUD and MDD	4.1%		Calculated; [18–20]
Effect on			
Rate of partner change^a^	+ 9.0%		[28]
HIV testing^a^	-70.0%		[2]
Linkage to care^a^	-65.0%		[8]
Adherence to ART^a^	-39.0%		[4]
***HIV transmission and risk behaviors***			
Annual probability of diagnosis (if undiagnosed)	20.2%		[29]
Annual probability of linkage to care after a positive HIV test			
Chronic stage (CD4 ≥ 200)	88.9%		[30, 31]
Advanced stage (CD4 < 200)	59.3%		[30, 31]
Probability of adherence to ART (proportion of doses taken as directed)	63.0%		[32–36]
Annual probability of ART regimen change in response to resistance	33.0%		[37]
Probability of virologic suppression			
Perfect adherence	95%		[38]
Average adherence	60%		[32–36, 38]
Sexual risk behaviors			
Annual rate of partner change	**Female**	**Male**	
Low risk	0.02	0.13	[39, 40]
Moderate risk	1.21	3.32	[40]
High risk	6.06	6.65	[39, 40]
Assortativity^b^ of sexual mixing by			
Partner change rate	0.32		[41]
Age	0.29		[41]
CASM	0.20		[41]
***Effectiveness of interventions***			
AUD screening and treatment			
On AUD	34.0%		[5, 21, 22]
On AUD and MDD (spillover)	+ 9.7%		[5, 21–24]
MDD screening and treatment			
On MDD	34.0%		[5, 23, 24]
On AUD and MDD (spillover)	+ 6.3%		[5, 21–24]
AUD and MDD screening and treatment			
On AUD	34.0%		[5, 21–23]
On MDD	34.0%		[5, 21–24]
On AUD and MDD	56.4%		[5, 21–24]
On AUD and MDD, spillover	+ 10.0%		[5, 21–24]
**Screening sensitivity and specificity**	**Sensitivity**	**Specificity**	
AUD	0.85	0.77	[5, 10, 11]
MDD	0.88	0.88	[5, 10, 11]
***Programmatic cost per intervention per person per year (2019 US $)***			
AUD screening	$ 3.94		Calculated; [5, 42–46]
AUD treatment	$15.80		Calculated; [5, 42–46]
MDD screening	$ 2.35		Calculated; [24, 45–49]
MDD treatment	$14.29		Calculated; [24, 45–49]
***Healthcare cost, price per person (US $)***			
Cost of a viral load test	$ 35.04		[50]
Cost of a CD4 test	$ 11.41		[51]
Annual cost of care	$ 277.86		[51]
Annual cost of hospitalization	$ 463.53		[51–53]
Annual cost of regimen 1	$ 150.36		[51, 54]

^a^Alcohol use disorder used as a proxy to estimate the effect

^b^Assortativity is the preference for connecting with other populations that are similar. It is represented on a scale of 0 to 1, where 0 represents random mixing regardless of characteristics and 1 represents completely non-random mixing with people of similar characteristics

*AUD* Alcohol use disorder, *MDD* Major depressive disorder, *ART* Antiretroviral therapy

Our baseline scenario simulated the following HIV interventions, which were chosen based on consultation with stakeholders in Zimbabwe, with coverage levels based on national health surveillance reports: voluntary male medical circumcision (VMMC), community testing, oral pre-exposure prophylaxis (PrEP), long-acting PrEP, differentiated care for treatment, defaulter tracing, HIV self-testing, and index tracing and testing. The effectiveness of interventions was obtained from published reports, and we made conservative assumptions about effect sizes to reflect the low adherence often observed in real-world settings (Additional file: Fig. S3). For example, only 30% of people starting oral PrEP were assumed to persist with treatment and sustain the preventive effect observed among the most adherent subgroups in randomized controlled trials [25]. While most estimates were from southern Africa, few of the estimates originated from Zimbabwe, so the generalizability of effectiveness estimates was reviewed by stakeholders before they informed model inputs.

Apart from the four policy scenarios, we also modeled a counterfactual scenario where CASM was eliminated from the population. While the counterfactual scenario was unrealistic, it provided insights into the maximum effect that future, improved CASM interventions could potentially have on HIV outcomes.

### Model calibration

Our model has been calibrated against four HIV outcomes (i.e., prevalence, incidence, number of people living with HIV [PWH], and HIV-related deaths,) using historical data from 1990 to 2020 (Additional file: Figs. S4–S6). We also calibrated HIV outcomes stratified by sex and age (Additional file: Figs. S5–S7). The data used for calibration come from a variety of government and international surveillance, including UNAIDS, World Population Prospects, and Zimbabwe Population-based HIV Impact Assessment (ZIMPHIA).

### CASM screening, treatment, and spillover

In the model, we assumed that everyone was screened for AUD and MDD using the AUDIT alcohol screener and PHQ-9 depression screener. The sensitivity and specificity of CASM screening instruments were based on the literature (Table 1). We chose screening instruments that were designed with global use in mind and, where possible, were previously translated to Shona [47, 55].

Those who screened positive for AUD would receive treatment involving motivational interviewing (MI) and cognitive behavioral therapy (CBT) from nurses [56]. Effectiveness of AUD treatment was based on published network meta-analysis of different types of nonpharmacologic talk therapies for the harmful use of alcohol [56], including RCTs of at least 12 weeks through 2022. The most effective AUD intervention was MI and CBT delivered in multiple face-to-face sessions, resulting in a mean reduction in AUDIT score of 5.0 (95% CI: 2.9–7.0). The mean reduction translates to a reduction in clinical harm above placebo of 34% (95% CI: 15–53%). The effectiveness estimate is similar to a South African randomized controlled trial, which found a 34% relative reduction in number of standard drinks [22], and to a separate study that found binge drinking reduced by 33% in an 8-h team-based intervention for employees in South Africa (Table 1) [57].

Those who screened positive for MDD would receive cognitive behavioral therapy from community health workers. Effectiveness of talk therapies for MDD was based on a randomized control trial of intensive CBT interventions for PWH in South Africa with clinical depression and lack of viral suppression. The study found an effect beyond placebo of approximately 34% (95% CI: 15–53%; Table 1) [24].

### Model structure

**Fig. 3:**
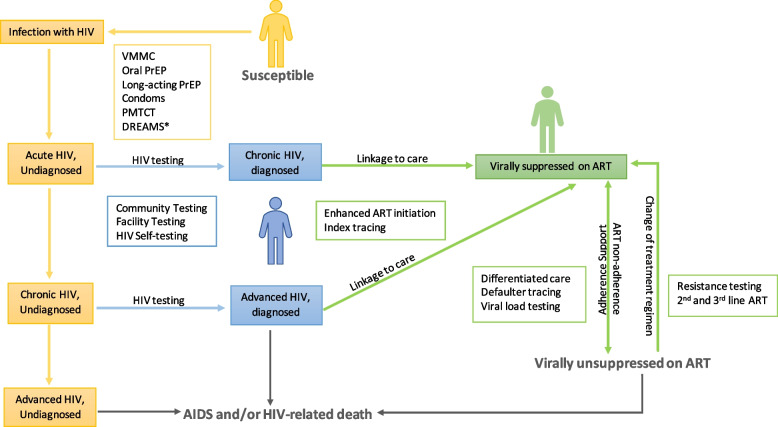
Model structure. Some interventions are included in the model structure, even if not being modeled in the current analysis. VMMC, voluntary medical male circumcision; PrEP, pre-exposure prophylaxis; PMTCT, prevention of mother to child transmission; ART, anti-retroviral therapy; DREAMS, Determined, Resilient, Empowered, AIDS-free, Mentored, and Safe. *Depending on how this intervention is configured, it may act at other sites too

HIV infection is represented through five sequential stages: *susceptible*, *acute HIV*, *chronic HIV*, *advanced HIV*, and *HIV-related death*. Individuals who newly contract HIV leave the susceptible stage and briefly pass through the acute stage before entering the chronic stage (Fig. 3). Those in the chronic stage move to the advanced stage at a progression rate that reflects the rate of CD4 decline in the absence of treatment (Table 1). Individuals can only have HIV-related death from the advanced stage. Beyond acute HIV infection, each of the HIV stages was further subdivided by diagnosis status (“yes” versus “no”), ART treatment status (“yes” versus “no,” and, if “yes,” further modified by adherence and retention-in-care), and viral load suppression (“yes” versus “no”). Virally unsuppressed individuals have the possibility of acquiring drug resistance and transitioning to second-line ART. The model distinguishes between wildtype and resistant HIV genotypes at the time of infection, and those who contracted resistant HIV could be virally unsuppressed on treatment even if fully adherent. Risk levels (i.e., low risk, medium risk, high risk) and risk types for men (i.e., MSM, non-MSM) impact HIV transmissibility through differing frequencies and/or risks of sexual contacts. Our model only considers vertical transmission and horizontal transmission through sex, as injection drug use is uncommon in Zimbabwe.

In our model, CASM interventions reduce the percentage of people with CASM conditions. The proportion of people with CASM conditions affects transmissibility because people with CASM conditions are more likely to engage in high-risk sexual activities and less likely to be adherent to ART, thereby raising their viral load. We assumed that a positive AUD screening would be followed by AUD treatment and that a positive MDD screening would similarly be followed by MDD treatment.

### Allocative efficiency analysis

Allocative efficiency analysis identifies “efficient frontiers,” strategies that maximize health gains for particular resource scenarios. Health gains considered were infections averted and quality-adjusted life years (QALYs) added. Costs considered included programmatic costs of interventions and healthcare-related costs. The programmatic cost for each intervention included fixed costs, such as durable equipment, facility, and rent; and variable costs, such as labor, supplies, drugs, and travels, where applicable. Healthcare-related costs included the cost of hospitalization, outpatient visits, laboratory tests (e.g., viral load, CD4), and ART. Costs were analyzed from a modified societal perspective and were standardized to the 2019 US dollar’s value.

We simulated cost, infections averted, and quality-adjusted life years (QALYs) gained for a base population of 15.3 million adults in 2020, through 2030 and beyond. We calculated incremental QALYs gained and additional infections averted till 2030 to evaluate Zimbabwe’s progress towards its 2030 EHE goal. In our primary analysis, we used a 3% discount rate for both costs and health outcomes.

### Sensitivity analysis

We performed five types of sensitivity analysis. First, we incorporated the phenomenon of “spillover.” Second, we varied the programmatic costs of less cost-effective interventions. Third, we varied the programmatic costs of cost-effective interventions. Fourth, we considered the impact of CASM interventions on non-HIV-related outcomes, in addition to their impact on HIV outcomes. Fifth, we conducted a probabilistic sensitivity analysis with varying cost and effectiveness inputs.

In the first sensitivity analysis, we considered “spillover,” where the successful treatment of AUD improves the probability of MDD remission, as well as the converse. Spillover probabilities were from causal inference analyses that emulated randomized control trials, using longitudinal data from the Veteran Aging Cohort Study (Table 1) [9]. Applying these results in our model, within a year of successful treatment of MDD, the probability of AUD remission that was attributable to MDD was 6.3%. Conversely, within a year of successful treatment of AUD, the probability of MDD remission that was attributable to AUD was 9.7% (Table 1; Fig. 2).

Second, we varied the cost assumptions of interventions that were less cost-effective (i.e., the ones that are not on the efficient frontier) because intervention costs may vary considerably as interventions are scaled. In particular, since screening and treatment of AUD and MDD involve similar levels of time and expertise, their costs may become similar at scale.

Third, we varied the cost assumptions of interventions that *are* on the efficient frontier. We conducted this analysis to identify the cost levels at which the most efficient CASM interventions become as cost-effective as other HIV interventions. We used long-acting PrEP as the point of comparison, which has an ICER of $2086 per QALY gained (unpublished data, Braithwaite RS).

Fourth, we considered the impact of CASM interventions on QALYs through improving non-HIV outcomes, on top of improving HIV outcomes. Screening and treatment for people with CASM conditions improve QALYs not just through HIV outcomes, but also through reducing the background mortality of those with CASM conditions (e.g., reducing road fatalities, reducing risks of cirrhosis). We incorporated the impact on background mortality to understand the full population health impact of CASM interventions.

Fifth, in probabilistic sensitivity analysis, we varied the costs and intervention effectiveness of AUD and MDD, with distributions that reflect cost variations and the 95% confidence interval of intervention effectiveness.

### Role of the funding sources

The funder of the study had no role in study design, data collection, data analysis, data interpretation, or writing of the report.

## Results

We describe our results in the following order: our projections for HIV incidence in 2030, our cost-effectiveness analysis results for cost per infections averted, and for cost per QALY gained. We then describe our sensitivity analysis results.

### Projections for HIV incidence

**Fig. 4:**
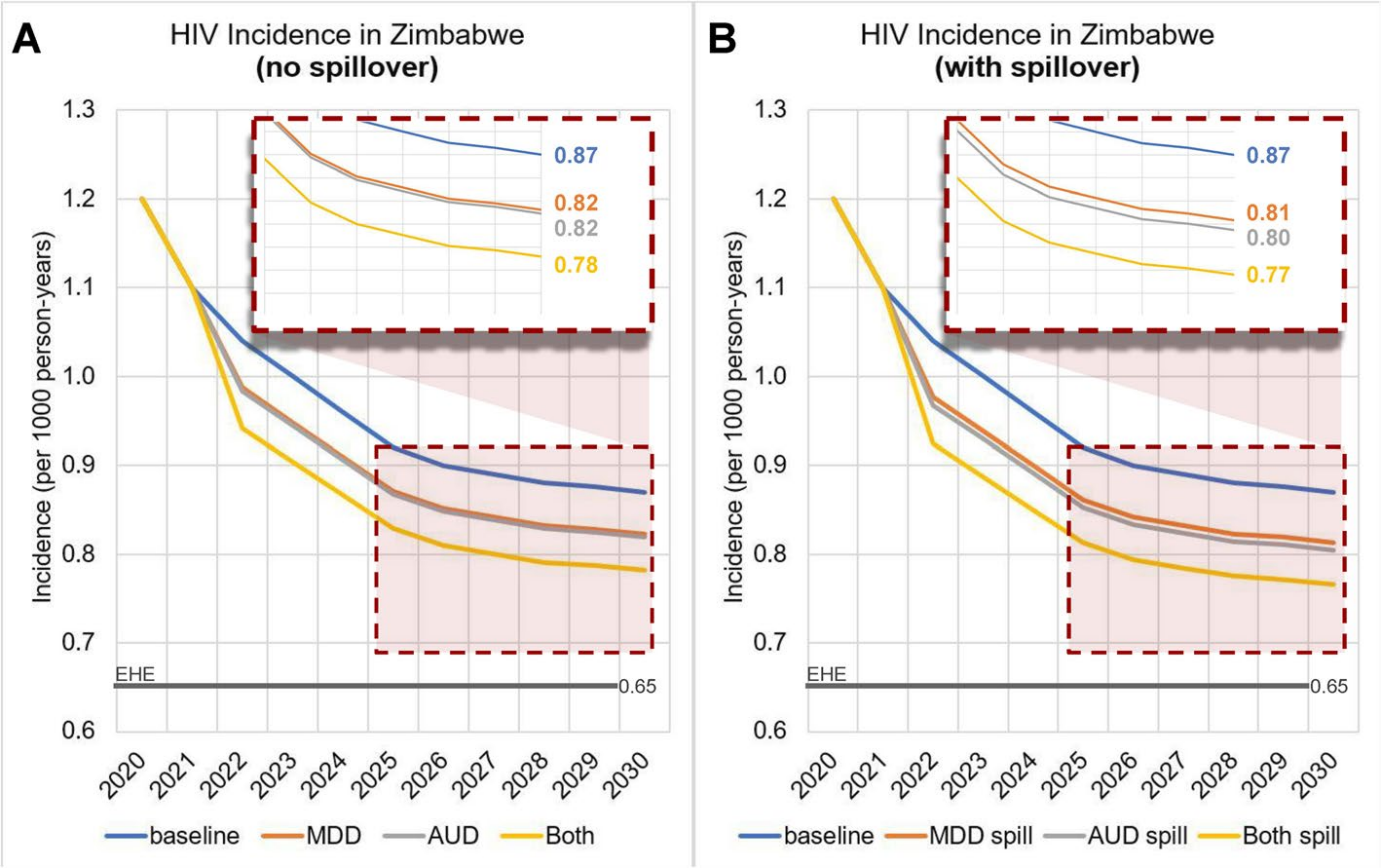
Projected HIV incidence in Zimbabwe over time. AUD, alcohol use disorder intervention; MDD, major depressive disorder intervention; AUD spill, alcohol use disorder intervention assuming spillover effects; MDD spill, major depressive disorder intervention assuming spillover effects

In 2030, without any CASM interventions, the baseline scenario would have an HIV incidence of 0.87 per thousand person-years. In the counterfactual scenario where CASM was eliminated, the incidence would be 0.69 per thousand person-years. Accordingly, CASM interventions thus have a maximal potential effect of reducing HIV incidence in 2030 by 21.1% (Fig. 4A).

Within this maximal reduction, MDD intervention alone produces 25.9% of the reduction, bringing the incidence in 2030 to 0.822 per thousand person-years. AUD intervention alone produces 27.9% of the maximal reduction, bringing the incidence in 2030 to 0.819 per thousand person-years. MDD and AUD interventions, when implemented together, produce approximately half of the maximal reduction (48.0%), reducing the incidence in 2030 to 0.78 per thousand person-years. However, this reduction alone is insufficient to reach Zimbabwe’s EHE goal of 0.65 per thousand person-years by 2030 (Fig. 4A).

### Cost-effectiveness analysis

**Fig. 5:**
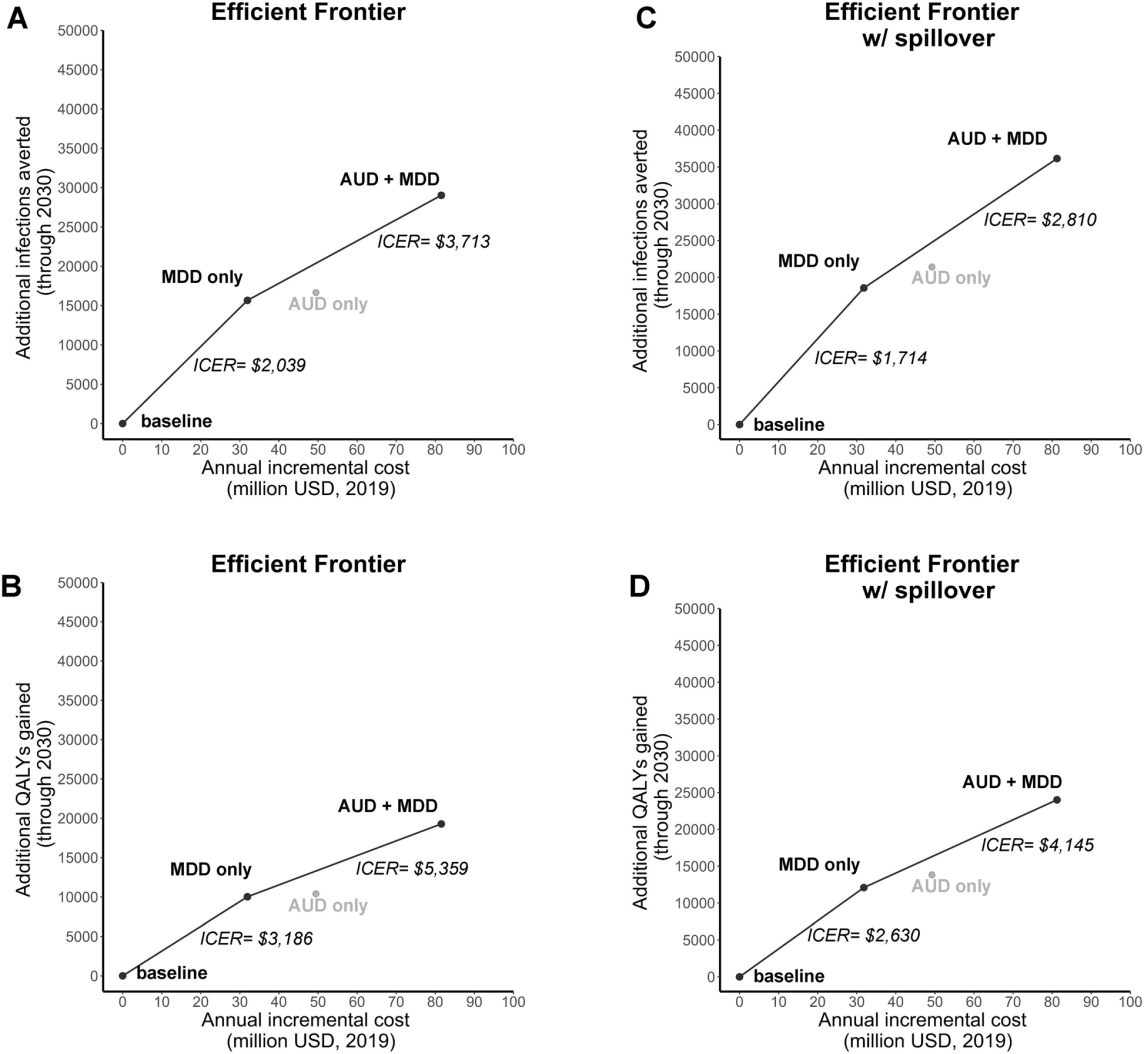
Efficient frontier in infections averted and QALYs gained, with varying spillover assumptions. Graph **A** shows the efficient frontier with infections averted as the effect, assuming no spillover. Graph **B** shows the efficient frontier with QALYs gained as the effect, assuming no spillover. Graph **C** shows the efficient frontier with infections averted as the effect, assuming there is spillover. Graph **D** shows the efficient frontier with QALYs gained as the effect, assuming there is spillover. AUD, alcohol use disorder intervention; MDD, major depressive disorder intervention; ICER, incremental cost-effectiveness ratio

In our cost-effectiveness analysis, implementing MDD intervention alone averts an additional 15,662 infections through 2030 and costs an additional USD$32 million per year, resulting in an incremental cost-effectiveness ratio (ICER) of $2039 per infections averted (Fig. 5A). The annual cost incurred from MDD intervention alone includes $33 million in programmatic costs and $383 million in healthcare costs.

Implementing AUD intervention alone is not as cost-effective. It averts an additional 16,663 infections through 2030 and costs an additional USD$49 million per year (Fig. 5A), resulting in an ICER of $2968 when compared to baseline. The annual cost incurred from AUD intervention alone includes $50 million in programmatic costs and $383 million in healthcare costs.

Implementing both AUD and MDD interventions averts an additional 29,022 infections through 2030 and incurs an additional USD$82 million per year, resulting in an ICER of $3713 per infections averted when compared to the adjacent strategy on the efficient frontier (i.e., MDD intervention; Fig. 5A). Implementing both results in an ICER of $2810 when compared to the baseline. The annual cost incurred from implementing both interventions includes $83 million in programmatic costs and $383 million in healthcare costs.

Results in QALYs suggest that MDD intervention alone will result in 10,025 additional QALYs through 2030, giving an ICER of $3186 per QALY gained (Fig. 5B). However, implementing AUD intervention alone is not as cost-effective. AUD intervention alone results in 10,404 additional QALYs through 2030, giving an ICER of $4753 compared to baseline. Implementing both AUD and MDD interventions will result in 19,282 additional QALYs through 2030, giving an ICER of $5359 per QALY gained when compared to the adjacent strategy on the efficient frontier (i.e., MDD intervention; Fig. 5B). Implementing both results in an ICER of $4229 when compared to the baseline.

### Sensitivity analysis

#### Sensitivity analysis of spillover effects

Assuming that spillover effects are in place, meaning that people with both MDD and AUD experience benefits not only from the treated condition, but also from the untreated condition, CASM interventions would have an even greater impact on HIV incidence. With spillover, MDD intervention alone captures 30.7% of the maximal reduction, as opposed to 25.9% when we assumed no spillover. AUD intervention alone captures 35.5%, as opposed to 27.6%. Both MDD and AUD, with spillover effects, would capture 56.6% of the maximal reduction, as opposed to 48.0% without spillover effects, thereby reducing incidence in 2030 from 0.87 per thousand person-years to 0.77 per thousand person-years (Fig. 4B).

Even with spillover effects in the model, AUD intervention alone is still not as cost-effective as MDD intervention alone or as implementing both interventions (Fig. 5C, D). This is true for results in infections averted and results in QALYs. With spillover effects, MDD intervention alone averts an additional 18,564 infections and results in 12,095 additional QALYs through 2030, resulting in an ICER of $1714 per infections averted (Fig. 5C), or $2630 per QALY gained (Fig. 5D).

AUD intervention alone averts an additional 21,418 infections and results in 13,829 additional QALYs through 2030, resulting in an ICER of $2299 per infections averted (Fig. 5C), or $3560 per QALY gained (Fig. 5D) when compared to baseline. AUD intervention alone is not on the efficient frontier even when spillover is assumed.

Implementing both AUD and MDD interventions averts an additional 36,149 infections and results in 24,017 additional QALYs through 2030. When compared to the adjacent strategy on the efficient frontier (i.e., MDD intervention; Fig. 5D), implementing both has an ICER of $2810 per infections averted (Fig. 5C), or $4145 per QALY gained (Fig. 5D). When compared to the baseline, implementing both has an ICER of $2247 per infections averted or $3382 per QALY gained.

#### Sensitivity analysis of cost inputs

When comparing CASM interventions to each other, AUD intervention is not as cost-effective as MDD intervention. Therefore, we varied the cost inputs of the AUD intervention to identify the threshold cost at which the AUD became as cost-effective as the MDD intervention. We found that for AUD intervention to be as cost-effective as MDD intervention, AUD intervention cost needs to be reduced by 25.8% from its original cost of $19.74 per person.

To compare cost-effective CASM interventions to other HIV interventions, we determined the threshold cost at which the MDD intervention became as cost-effective as a current priority for scaling long-acting PrEP. We found that for MDD intervention to be as cost-effective as long-acting PrEP, the cost of MDD intervention would need to be reduced by 22.6% from its original cost of $16.64 per person.

#### Sensitivity analysis of non-HIV related outcomes

When we considered improvements in non-HIV-related outcomes in addition to improvements in HIV outcomes, we found that the ICER for MDD intervention alone reduced from $2630 per QALY gained to $2315. The ICER for implementing both MDD and AUD interventions reduced from $4145 per QALY gained to $3678, when compared to the adjacent strategy on the efficient frontier (i.e., MDD intervention alone). When compared to the baseline scenario, the ICER for implementing both interventions reduced from $3382 per QALY to $2989 per QALY.

#### Probabilistic sensitivity analysis

**Fig. 6:**
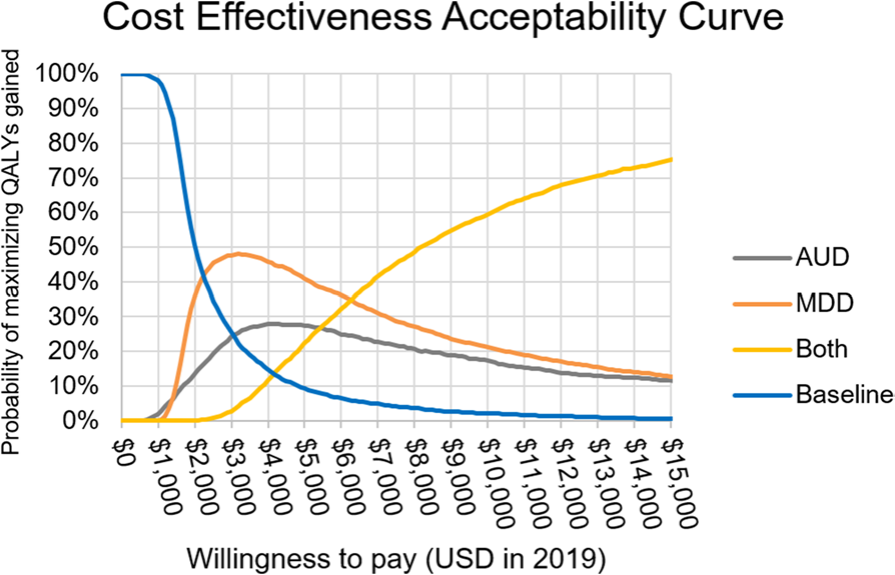
Cost-effectiveness acceptability curve (CEAC). Results from the probabilistic sensitivity analysis. AUD, alcohol use disorder intervention; MDD, major depressive disorder intervention; QALY, quality-adjusted life year

In our probabilistic sensitivity analysis, we found that MDD intervention alone has the highest probability of maximizing QALYs gained with willingness to pay (WTP) between $2200 and $6400 per QALY gained. At WTP below $2200 per QALY gained, not implementing anything (i.e., the baseline scenario) is the most favorable strategy, because greater health gains could be obtained with alternative HIV interventions. At WTP above $6400 per QALY gained, it is more favorable to implement both AUD and MDD interventions (Fig. 6).

## Discussion

Our modeling results suggest that incorporating CASM interventions in HIV care can reduce Zimbabwe’s HIV incidence over the next 10 years. However, that reduction in incidence alone is insufficient for Zimbabwe to reach its EHE goal by 2030 without other programmatic changes. When AUD and MDD interventions were implemented and their spillover effects were considered, we estimated that Zimbabwe can close 40% of the gap between its EHE goal (0.65 per thousand person-years) and the baseline scenario (0.87 per thousand person-years) in 2030. Extensive literature has supported the link between CASM conditions, such as AUD and MDD, and HIV outcomes [1–5]. Yet, few modeling studies have explored the potential for CASM interventions to contribute to national HIV epidemiology control goals. Our study supported a role for screening and treating CASM interventions among Zimbabwe’s HIV population to contribute to Zimbabwe’s EHE goal.

Our study was the first to illuminate the full potential impact that future improved CASM interventions can have on Zimbabwe’s HIV incidence. Our projection showed that in the hypothetical situation where CASM conditions were eliminated, HIV incidence can reduce by 21% from the baseline scenario, where no CASM interventions were implemented. While this hypothetical scenario is unrealistic from a policy perspective, it demonstrates the potential impact on population health that improved CASM interventions can have. Our results are consistent with past modeling studies focusing on AUD in other Southern African contexts. A prior modeling study in Kenya concluded that around 13.0% to 16.5% of new HIV infections were attributable to unhealthy alcohol use [58]. Our study attributed 21% of HIV incidence in 2030 as potentially preventable with both AUD and MDD interventions. An agent-based modeling study in South Africa also found that a multi-session alcohol counseling intervention could reduce HIV incidence by 1.2% over five years, compared to a status quo scenario [59]. Our study projected a larger reduction in Zimbabwe, where AUD intervention alone could reduce HIV incidence by 5.8% over 10 years, compared to our baseline scenario. The difference between our projections could be attributed to differences in model assumptions and inputs. The agent-based model only represented the link between AUD and sexual risk behavior. Our model incorporated the impact of AUD beyond sexual risk behavior, particularly on treatment adherence and viral load suppression. Our model also assumed a slightly higher effectiveness of AUD intervention (34% reduction vs. 29% reduction). Spillover assumptions have some, albeit modest, influence on how much CASM interventions change HIV outcomes.

Our results suggested that MDD intervention is more cost-effective than AUD intervention. The costs of screening and treating MDD are lower than those of AUD, primarily because MDD intervention is typically administered by community health workers, whereas AUD intervention is administered by nurses in Southern Africa. Reducing the cost of the AUD intervention from $19.74 per person to $14.65 per person would make it as cost-effective as the MDD intervention. Since practices for treating AUD and MDD are sometimes similar, there may be potential to reduce the labor cost of the AUD intervention, which may improve its efficiency enough to become a preferred strategy. Additionally, if AUD and MDD were simultaneously targeted, the same programmatic resources may be leveraged for both goals, further improving their cost-effectiveness.

It is important to note that at an ICER of $2630 per QALY gained considering spillover effects, MDD intervention comes close but is not as cost-effective as expanding long-acting PrEP, which is a current HIV prevention priority in Zimbabwe and has an ICER of $2086 per QALY gained. However, reducing the cost of MDD intervention by 22.6% would make it as cost-effective as long-acting PrEP.

Our estimations for the cost-effectiveness of MDD align closely with Zimbabwe’s National HIV and AIDs Strategic Plan (ZNASP IV). The Ministry of Health and Childcare has identified mental health screening and provision as a strategy in its HIV key population program, which targets high-risk groups such as sex workers and prisoners, among others. Targeting MDD screening and treatment at high-risk population may further improve MDD intervention’s cost-effectiveness. The goal, according to the ZNASP IV, is to screen and diagnose as many as 90% of PWH for mental health by 2025. There is currently no programmatic data on the number of PWH screened and diagnosed for mental health, nor is it standard practice to screen for CASM conditions in HIV clinics in Zimbabwe. Improving health surveillance capacity for PWH with CASM conditions could allow Zimbabwe to further tap into the potential impact of CASM.

Our probabilistic sensitivity analysis suggests that the favored policy decision is highly dependent on willingness to pay (WTP). Zimbabwe’s WTP lie between USD$1422 to $4266 per QALY gained, if we were to reference the World Health Organization’s recommendation to take one to three times the gross domestic product (GDP) per capita. MDD intervention alone is most favorable for most WTPs according to this method. However, recent literature has recommended WTP thresholds that considered health opportunity costs, with Zimbabwe’s WTP estimated at a much lower range of USD$41 to $874 [60]. While these recommendations are inconsistent with current programmatic priorities such as expanding long-acting PrEP, they would imply that implementing neither MDD nor AUD intervention (e.g., the baseline scenario) is the most favored policy decision.

Our study has important limitations. We opted to focus on AUD and MDD interventions, based on anecdotal evidence of the relatively low prevalence of other CASM conditions in Zimbabwe. However, as a result, we were not able to capture spillover effects across CASM conditions other than MDD and AUD, such as substance use disorder, tobacco use disorder, anxiety disorder, and chronic pain. As such, our model potentially underestimated the true burden of disease that more comprehensive CASM screenings and treatments could avert. While our model incorporated screening sensitivity and specificity, we assumed perfect diagnosis for both AUD and MDD, a choice we made to simplify the model. Future studies could benefit from incorporating diagnostic specificity and sensitivity. We also assumed that everyone diagnosed of AUD and MDD would seek and receive treatment, which may be idealistic given that cultural attitudes around AUD and MDD may discourage individuals with those conditions from seeking treatment. Future studies can also explore how targeting high-risk populations, such as sex workers and men who have sex with men (MSM), could impact the cost-effectiveness of CASM interventions. CASM interventions, when targeted at high-risk populations, may have the potential to fall within Zimbabwe’s WTP. Further, given that CASM conditions often have overlapping treatment methods, future studies could also explore using transdiagnostic interventions as a potential way to lower the cost of CASM treatment.

## Conclusions

In conclusion, while CASM interventions on their own are insufficient to enable Zimbabwe to reach its EHE 2030 goal, they could play an important role by closing nearly half of the gap between that goal and current projections. Further, if modest cost reductions of approximately 25% in CASM interventions were possible while preserving their effectiveness, CASM interventions would attain value-parity with emerging programmatic priorities such as scaling long-acting PrEP.

## Supplementary Information

Supplementary Material 1: FigS1. Model structure for HIV progression. FigS2. Model inputs. FigS3. Intervention inputs. FigS4. Calibration results by total population. FigS5. Calibration results stratified by age and sex. FigS6. Prevalence calibration results. FigS7. Incidence calibration results.

## Data Availability

All used in the study for model parameterization are publicly available (see Table 1, Additional file: Figs. S2 and S3) and data for model calibration is available from the UNAIDS, World Population Prospects, and the Zimbabwe Population-based HIV Impact Assessment (ZIMPHIA).

## Abbreviations

EHE: Ending the HIV Epidemic goal
CASM: Constellation of alcohol, substance, and mood-related conditions
AUD: Alcohol use disorder
MDD: Major depressive disorder
ICER: Incremental cost-effectiveness ratio
WTP: Willingness-to-pay
MSM: Men who have sex with men
PWH: People with HIV
PrEP: Pre-exposure prophylaxis
GDP: Gross domestic product
USD: United States dollar
HIV: Human immunodeficiency virus
ART: Antiretroviral therapy
AUDIT: Alcohol Use Disorders Identification Test
PHQ-9: Patient Health Questionnaire
QALY: Quality-adjusted life years
VMMC: Voluntary medical male circumcision
MI: Motivational interviewing
CHW: Community health worker
CBT: Cognitive behavioral therapy
ZNASP IV: Zimbabwe’s National HIV and AIDs Strategic Plan

## Data availability

No datasets were generated or analysed during the current study.
