# Supplementary material for: Cost-effectiveness of screening and treating alcohol use and depression among people living with HIV in Zimbabwe: a mathematical modeling study
Source: BMC Med. 2024 Oct 21;22:481. doi: 10.1186/s12916-024-03674-8 (PMC11492560; doi:10.1186/s12916-024-03674-8)
Supplement: Supplementary file 1 — Supplementary Material 1: FigS1. Model structure for HIV progression. FigS2. Model inputs. FigS3. Intervention inputs. FigS4. Calibration results by total population. FigS5. Calibration results stratified by age and sex. FigS6. Prevalence calibration results. FigS7. Incidence calibration results. [file 12916_2024_3674_MOESM1_ESM.pdf]

Additional File

Technical appendix for "Cost-effectiveness of screening and treating alcohol use and depression among people living with HIV in Zimbabwe: a mathematical modeling study"

We created a compartmental transmission model that simulated the effects of CASM screening and treatment on HIV transmission and life expectancy. Here, CASM stands for Constellation of Alcohol, Substance, and Mood-related conditions, and includes the following six conditions: alcohol use disorder, major depressive disorder, anxiety disorder, opioid use disorder, stimulant use disorder, and smoking.

Population

The modeled population is structured by age, gender, sexual preferences, risks, and CASM status.

Age

The model includes children (age 0-14); adolescents and young people (age 15-24); young adults (age 25-34); adults (age 35-64); and older adults (age 65 and older).

Gender and sexual preference

The modeled population is divided into women and men of equal proportions. The adults groups covering ages 15-64 are the only groups assumed to be sexually active, and men in these groups are further divided into heterosexual men and MSM.

Risk

Women and heterosexual men can be in one of four risk strata: no risk (not sexually active), low risk (long-term, monogamous partnerships), moderate risk (non-monogamous partnerships), and high risk (multiple simultaneous non-monogamous partnerships, e.g. transactional sex).

CASM Conditions

Adults ages 15-64 can additionally be classified as having CASM status. Of the six CASM conditions listed at the start of the appendix, we focus on alcohol use disorder and major depressive disorder in this work. The parameters governing adults with CASM status are modified such that the average rates of HIV diagnosis, linkage to care, and adherence to ART are lower, and engagement in risky sexual behaviors is more likely.

Simulation

Groups in the model start out uninfected and, once infected with HIV, progress through the stages of acute HIV infection, chronic HIV infection, and advanced HIV. We assume that HIV-related death occurs only from the advanced HIV stage. Our modeled care cascade divides all HIV-positive people into undiagnosed, diagnosed on successful ART with a non-transmissible viral load, and diagnosed on unsuccessful ART with a transmissible viral load. For people with wildtype HIV, therapy is assumed to be first-line and the viral load is initially successfully suppressed, but some proportion of people then lose viral suppression due to non-adherence or treatment interruption. We allow for re-initiation of first-line therapy after this loss of viral suppression, but in the meantime the unsuppressed virus can acquire drug resistance. People with drug-resistant HIV are unresponsive to first-line therapy and remain virally unsuppressed unless and until they are switched to subsequent lines, after which we assume their viral load is successfully suppressed. People can also die of HIV-related causes from the advanced HIV stage or if they are virally unsuppressed.

A detailed pictorial description of the simulation is depicted in **Fig. S1** with relevant model inputs summarized in **Fig. S2**.

HIV transmission

The HIV transmission routes we consider in our model are heterosexual sexual transmission between adults, same-sex sexual transmission between MSM, and mother-to-child transmission. For brevity, we will exclude the simpler equations governing HIV transmission in children (ages 0-14) and older adults (ages 65+), as we assume no sexual HIV transmission occur among these two groups.

The adult HIV-negative population, $X(t)$, is divided into subgroups by age group (i), with $i = 1$ denoting adolescents (age 15-24), $i = 2$ young adults (age 25-34), and $i = 3$ adults (age 35-64); sexual risk group (j), with $j = 1$ denoting low risk, $j = 2$ moderate risk, and $j = 3$ high risk; and CASM status (k), with $k = 1$ denoting the absence of CASM conditions and $k = 2$ their presence.

The HIV-positive adult population, $Y(t)$, has the same demographic and sexual behavior indices; additional indices represent resistance status (r), with $r = 1$ denoting wildtype HIV and $r = 2$ drug-resistant HIV, and infection stage (s), with $s = 1$ denoting acute HIV, $s = 2$ chronic HIV with no knowledge of status, $s = 3$ advanced HIV with no knowledge of status, $s = 4$ chronic HIV with knowledge of status, $s = 5$ advanced HIV with knowledge of status, $s = 6$ unsuccessful viral suppression on ART, and $s = 7$ successful viral suppression on ART.

Equations governing HIV-negative groups

HIV-negative compartments ($X_{i,j,k}$) change over time according to the equation

$$\frac{dX_{i,j,k}(t)}{dt} = \overbrace{\alpha_{i-1}X_{i-1,j,k}(t)}^{\text{aging in}} - \overbrace{\alpha_i X_{i,j,k}(t)}^{\text{aging out}} - \overbrace{\lambda_{i,j,k}(t)X_{i,j,k}(t)}^{\text{HIV infection}}$$

where the parameter α_i denotes the rate of aging out of age group i and the function $\lambda_{i,j,k}(t)$ describes the average force of HIV infection experienced by a group with demographic and behavior characteristics i, j, and k.

Equations governing HIV-positive groups who are not in care

HIV-positive groups who are not yet in care (disease states $s = 1$ to $s = 5$) are governed by the following equations:

$$\overbrace{\frac{dY_{i,j,k,r,s=1}(t)}{dt}}^{\text{acute HIV}} = \overbrace{\alpha_{i-1}Y_{i-1,j,k,r,s=1}(t) - \alpha_i Y_{i,j,k,r,s=1}(t)}^{\text{aging}} + \overbrace{\lambda_{i,j,k,r}X_{i,j,k}(t)}^{\text{HIV infection}} - \overbrace{\gamma_1 Y_{i,j,k,r,s=1}(t)}^{\text{progression}}$$

$$\overbrace{\frac{dY_{i,j,k,r,s=2}(t)}{dt}}^{\substack{\text{chronic HIV}\\ \text{with no knowledge}\\ \text{of status}}} = \overbrace{\alpha_{i-1}Y_{i-1,j,k,r,s=2}(t) - \alpha_i Y_{i,j,k,r,s=2}(t)}^{\text{aging}} + \overbrace{\gamma_1 Y_{i,j,k,r,s=1}(t) - \gamma_2 Y_{i,j,k,r,s=2}(t)}^{\text{progression}} - \overbrace{\kappa_{i,j,k,s=2}Y_{i,j,k,r,s=2}(t)}^{\text{HIV testing}}$$

$$\overbrace{\frac{dY_{i,j,k,r,s=3}(t)}{dt}}^{\substack{\text{advanced HIV}\\ \text{with no knowledge}\\ \text{of status}}} = \overbrace{\alpha_{i-1}Y_{i-1,j,k,r,s=3}(t) - \alpha_i Y_{i,j,k,l,s=3}(t)}^{\text{aging}} + \overbrace{\gamma_2 Y_{i,j,k,r,s=2}(t)}^{\text{progression}} - \overbrace{\kappa_{i,j,k,s=3}Y_{i,j,k,r,s=3}(t)}^{\text{HIV testing}} - \overbrace{\zeta Y_{i,j,k,r,s=3}(t)}^{\substack{\text{HIV-related}\\ \text{death}}}$$

$$\overbrace{\frac{dY_{i,j,k,r,s=4}(t)}{dt}}^{\substack{\text{chronic HIV}\\ \text{with knowledge}\\ \text{of status}}} = \overbrace{\alpha_{i-1}Y_{i-1,j,k,r,s=4}(t) - \alpha_i Y_{i,j,k,r,s=4}(t)}^{\text{aging}} - \overbrace{\gamma_2 Y_{i,j,k,r,s=4}(t)}^{\text{progression}} + \overbrace{\kappa_{i,j,k,s=2}Y_{i,j,k,r,s=2}(t)}^{\text{HIV testing}} - \overbrace{\kappa_{i,j,k,s=4}Y_{i,j,k,r,s=4}(t)}^{\text{linkage to care}}$$

$$\overbrace{\frac{dY_{i,j,k,r,s=5}(t)}{dt}}^{\substack{\text{advanced HIV}\\\text{with knowledge}\\\text{of status}}} = \overbrace{\alpha_{i-1}Y_{i-1,j,k,r,s=5}(t) - \alpha_i Y_{i,j,k,r,s=5}(t)}^{\text{aging}} + \overbrace{\gamma_2 Y_{i,j,k,r,s=4}(t)}^{\text{progression}}$$
$$+ \overbrace{\kappa_{i,j,k,s=3}Y_{i,j,k,r,s=3}(t)}^{\text{HIV testing}} - \overbrace{\kappa_{i,j,k,s=5}Y_{i,j,k,r,s=5}(t)}^{\text{linkage to care}} - \overbrace{\zeta Y_{i,j,k,r,s=5}(t)}^{\substack{\text{HIV-related}\\\text{death}}}$$

where the function $\lambda_{i,j,k,r}$ denotes the force of HIV infection experienced by the HIV-susceptible group $X_{i,j,k}$, with the resistance status r transmitted from the infected group from whom the infection has come (such that the total force of infection experienced by susceptible groups is $\sum_{r=1}^{R} \lambda_{i,j,k,r} = \lambda_{i,j,k}$). The parameters γ_1 and γ_2 denote rates of progression from acute to chronic HIV and from chronic to advanced HIV, respectively; $\kappa_{i,j,k,s}$ denotes the rate of HIV testing and linkage to care when asymptomatic ($s = 2$ and $s = 4$, respectively) or symptomatic ($s = 3$ and $s = 5$, respectively); and ζ denotes the rate of HIV-related death, which we assume is negligible except from the advanced stage.

Equations governing HIV-positive groups who are in care

We divide HIV-positive people who are in care into those with wildtype ($r = 1$) or drug-resistant ($r = 2$) HIV who are unsuccessfully ($s = 6$) or successfully ($s = 7$) virally suppressed on ART. These groups are governed by the following equations:

$$\overbrace{\frac{dY_{i,j,k,r=1,s=6}(t)}{dt}}^{\substack{\text{wildtype HIV,}\\\text{unsuccessful}\\\text{viral suppression}\\\text{on ART}}} = \overbrace{\alpha_{i-1}Y_{i-1,j,k,r=1,s=6}(t) - \alpha_i Y_{i,j,k,r=1,s=6}(t)}^{\text{aging}} - \overbrace{\zeta Y_{i,j,k,r=1,s=6}(t)}^{\substack{\text{HIV-related}\\\text{death}}}$$
$$+ \overbrace{\frac{1}{\nu_{i,j,k}}Y_{i,j,k,r=1,s=7}(t)}^{\text{loss of viral suppression}} - \overbrace{\xi Y_{i,j,k,r=1,s=6}(t)}^{\substack{\text{acquisition of}\\\text{drug resistance}}}$$

$$\overbrace{\frac{dY_{i,j,k,r=1,s=7}(t)}{dt}}^{\substack{\text{wildtype HIV,}\\\text{successful}\\\text{viral suppression}\\\text{on ART}}} = \overbrace{\alpha_{i-1}Y_{i-1,j,k,r=1,s=7}(t) - \alpha_i Y_{i,j,k,r=1,s=7}(t)}^{\text{aging}}$$
$$+ \overbrace{\kappa_{i,j,k,s=4}Y_{i,j,k,r=1,s=4}(t) + \kappa_{i,j,k,s=5}Y_{i,j,k,r=1,s=5}(t)}^{\text{linkage to care}}$$
$$- \overbrace{\frac{1}{\nu_{i,j,k}}Y_{i,j,k,r=1,s=6}(t)}^{\text{loss of viral suppression}}$$

$$\overbrace{\frac{dY_{i,j,k,r=2,s=6}(t)}{dt}}^{\substack{\text{drug-resistant HIV,}\\\text{unsuccessful}\\\text{viral suppression}\\\text{on ART}}} = \overbrace{\alpha_{i-1}Y_{i-1,j,k,r=2,s=6}(t) - \alpha_i Y_{i,j,k,r=2,s=6}(t)}^{\text{aging}} - \overbrace{\zeta Y_{i,j,k,r=2,s=6}(t)}^{\substack{\text{HIV-related}\\\text{death}}}$$
$$+ \overbrace{\kappa_{i,j,k,s=4}Y_{i,j,k,r=2,s=4}(t) + \kappa_{i,j,k,s=5}Y_{i,j,k,r=2,s=5}(t)}^{\text{linkage to care}}$$
$$+ \overbrace{\xi Y_{i,j,k,r=1,s=6}(t)}^{\substack{\text{acquisition of}\\\text{drug resistance}}} - \overbrace{\pi Y_{i,j,k,r=2,s=6}(t)}^{\text{therapy line switching}}$$

$$\overbrace{\frac{dY_{i,j,k,r=2,s=7}(t)}{dt}}^{\substack{\text{drug-resistant HIV,}\\\text{successful}\\\text{viral suppression}\\\text{on ART}}} = \overbrace{\alpha_{i-1}Y_{i-1,j,k,r=2,s=7}(t) - \alpha_i Y_{i,j,k,r=2,s=7}(t)}^{\text{aging}} + \overbrace{\pi Y_{i,j,k,r=2,s=6}(t)}^{\text{therapy line switching}}$$

where $\nu_{i,j,k}$ denotes the average duration of adherence to ART (dependent on subgroup characteristics), ξ denotes the rate at which HIV acquires drug resistance in people who are not virally suppressed on ART, and π denotes the rate of switching from first-line to second-line ART (or subsequent lines) to compensate for drug resistance.

It is assumed that people with drug-resistant HIV ($r = 2$) can only achieve viral suppression by being switched onto second-line ART (or subsequent lines). A further simplifying assumption is that the criterion for therapy line switching accurately reflects HIV resistance status among people who are on ART but not virally suppressed.

Heterosexual mixing dynamics

HIV infection can be heterosexual—either to susceptible women from HIV-positive men or to susceptible men from HIV-positive women—or homosexual (to susceptible men who have sex with men from HIV-positive men who have sex with men).

We first denote the preferred partner change rate specific to women and to (heterosexual) men as $c_{w;i,j,k}$ (or $c_{w;i',j',k'}$ if from the perspective of partners) and $c_{m;i,j,k}$ (or $c_{m;i',j',k'}$ if from the perspective of partners), respectively. The preferred partner change rate, taking women for example, is as follows:

$$c_{w;i,j,k} = c^*_{w;j}\mu_{1;i}\mu_{2;k} \tag{1}$$

where $c^*_{w;j}$ denotes the baseline partner change rate by gender (in this case, women w) and risk group j (such that men on average prefer a higher partner change rate than women, with the exception of female sex workers; and higher risk groups of both genders prefer a higher partner change rate than lower risk groups),

$\mu_{2;i}$ denotes a modifier of the partner change rate dependent on age group i (such that people may change partners more slowly as they age into older age groups), and $\mu_{2;k}$ denotes a modifier of partner change rate dependent on CASM status k (such that people with CASM characteristics may change partners more frequently). This design of the partner change rate was chosen in order to reflect the way it has been studied in the literature.

Let $\delta_{1;i,i'}$ denote the Kronecker delta with respect to age (whereby $\delta_{1;i,i'} = 1$ if the subject and partner are in the same age group, $i = i'$; and $\delta_{1;i,i'} = 0$ if $i \neq i'$); $\delta_{2;j,j'}$ with respect to risk (whereby $\delta_{2;j,j'} = 1$ if the subject and partner are in the same risk group, $j = j'$; and $\delta_{2;j,j'} = 0$ if $j \neq j'$); and $\delta_{3;k,k'}$ with respect to CASM status (whereby $\delta_{3;k,k'} = 1$ if the subject and partner have the same CASM status, $k = k'$; and $\delta_{3;k,k'} = 0$ if $k \neq k'$).

Let ϵ_1 denote assortativity of sexual mixing between subjects and partners by age, ϵ_2 by risk, and ϵ_3 by CASM status. We define these ϵ's as ranging from entirely proportionate when $\epsilon = 0$ to entirely assortative when $\epsilon = 1$. (We do not consider disassortative mixing, $\epsilon < 0$, as this is likely to be unrealistic for the types of mixing we are interested in.)

Mixing is designed such that for entirely proportionate mixing ($\epsilon_1 = \epsilon_2 = \epsilon_3 = 0$), the contact pattern multiplies out to be the number of contacts offered by each class (i.e. by each partner age i', risk level j', and CASM status k') divided by all possible contacts (the sum over all i', j', and k').

The preferred mixing matrix for female subjects of classes i, j, and k with male partners of classes i', j', and k' is denoted as $M^*_{w;i,j,k,i',j',k'}$. This is given by:

$$M^*_{w;i,j,k,i',j',k'} = c_{w;i,j,k} \left(\epsilon_1 \delta_{1;i,i'} + (1-\epsilon_1) \frac{\sum_{j'=1}^{J} \sum_{k'=1}^{K} C_{m;i',j',k'}}{\sum_{i'=1}^{I} \sum_{j'=1}^{J} \sum_{k'=1}^{K} C_{m;i',j',k'}} \right) \left(\epsilon_2 \delta_{2;j,j'} + (1-\epsilon_2) \frac{\sum_{k'=1}^{K} C_{m;i',j',k'}}{\sum_{j'=1}^{J} \sum_{k'=1}^{K} C_{m;i',j',k'}} \right) \left(\epsilon_3 \delta_{3;k,k'} + (1-\epsilon_3) \frac{C_{m;i',j',k'}}{\sum_{k'=1}^{K} C_{m;i',j',k'}} \right)$$

where $C_{m;i',j',k'}$ is the total number of contacts offered by male partners of class i', j', and k', calculated as follows:

$$C_{m;i',j',k'} = c_{m;i',j',k'} \left(X_{m;i',j',k'} + \sum_{r'=1}^{R} \sum_{s'=1}^{S} Y_{m;i',j',k',r',s'} \right)$$

The preferred mixing matrix for male subjects of classes i, j, and k with male partners of classes i', j', and

k' is denoted as $M^*_{m;i,j,k,i',j',k'}$. This is given by:

$$M^*_{m;i,j,k,i',j',k'} = c_{m;i,j,k} \left(\epsilon_1 \delta_{1;i,i'} + (1-\epsilon_1) \frac{\sum_{j'=1}^{J} \sum_{k'=1}^{K} C_{w;i',j',k'}}{\sum_{i'=1}^{I} \sum_{j'=1}^{J} \sum_{k'=1}^{K} C_{w;i',j',k'}} \right) \left(\epsilon_2 \delta_{2;j,j'} + (1-\epsilon_2) \frac{\sum_{k'=1}^{K} C_{w;i',j',k'}}{\sum_{j'=1}^{J} \sum_{k'=1}^{K} C_{w;i',j',k'}} \right) \left(\epsilon_3 \delta_{3;k,k'} + (1-\epsilon_3) \frac{C_{w;i',j',k'}}{\sum_{k'=1}^{K} C_{w;i',j',k'}} \right)$$

where $C_{w;i',j',k'}$ is the total number of contacts offered by female partners of class i', j', and k', calculated as follows:

$$C_{w;i',j',k'} = c_{w;i',j',k'} \left(X_{w;i',j',k'} + \sum_{r'=1}^{R} \sum_{s'=1}^{S} Y_{w;i',j',k',r',s'} \right)$$

We then adjust for any imbalances in preferred mixing patterns between the sexes. Letting B denote the degree of imbalance between female subject and male partner preferences, we have:

$$B_{i,j,k,i',j',k'} = \frac{\left(X_{m;i',j',k'} + \sum_{r'=1}^{R} \sum_{s'=1}^{S} Y_{m;i',j',k',r',s'} \right) M^*_{m;i',j',k',i,j,k}}{\left(X_{w;i,j,k} + \sum_{r=1}^{R} \sum_{s=1}^{S} Y_{w;i,j,k,r,s} \right) M^*_{w;i,j,k,i',j',k'}}$$

The preferred mixing matrices are then adjusted according to this imbalance to obtain the final mixing matrices:

$$M_{w;i,j,k,i',j',k'} = M^*_{w;i,j,k,i',j',k'} B^{\theta}_{i,j,k,i',j',k'}$$
$$M_{m;i',j',k',i,j,k} = M^*_{m;i',j',k',i,j,k} B^{\theta-1}_{i,j,k,i',j',k'}$$

where θ is the degree of compromise between the sexes in the choosing of sexual partnerships. For simplicity we assume $\theta = 0.5$, meaning that men and women have equal control over partnerships.

Mixing dynamics of MSM

For homosexual partnerships, we make the simplifying assumption that MSM prefer to change male partners on average at the same rate as heterosexual men of moderate risk ($j = 2$) change female partners. The

homosexual partner change rate is thus given by:

$$c_{\mathrm{MSM};i,k} = c^*_{m;j=2}\mu_{1;i}\mu_{2;k}\rho$$

where $c^*_{m;j=2}$ is the baseline partner change rate for moderate-risk heterosexual men; $\mu_{1;i}$ and $\mu_{2;k}$ are modifying factors reflecting variation in preferences by age i and CASM status k as above. Because some MSM also partner with women, we specify ρ as the preferred ratio of male partners over female partners.

We make a further simplifying assumption that homosexual mixing is proportionate; i.e., random by age and CASM status in proportion to the contacts available. The resulting mixing matrix is given by:

$$M_{\mathrm{MSM};i,k,i',k'} = c_{\mathrm{MSM};i,k}c_{\mathrm{MSM};i',k'}\frac{X_{\mathrm{MSM};i',k'} + \sum_{r'=1}^{R}\sum_{s'=1}^{S} Y_{\mathrm{MSM};i',k',r',s'}}{\sum_{i'=1}^{I}\sum_{k'=1}^{K}\left(X_{\mathrm{MSM};i',k'} + \sum_{r'=1}^{R}\sum_{s'=1}^{S} Y_{\mathrm{MSM};i',k',r',s'}\right)}$$

For same-sex partnerships, it is not necessary to balance the mixing matrix.

Force of HIV infection

The force of infection experienced by susceptible subjects from HIV-positive partners, $\Lambda_{i,j,k}$, is calculated by multiplying the mixing matrix by the HIV prevalence among the partners contacted and the transmission probability by resistance status and disease stage.

We assume that the force of HIV infection experienced by MSM in heterosexual partnerships with women is the same as that experienced by heterosexual men of moderate risk.

The force of infection (by drug-resistance status) experienced by women from sexual partnerships with men is given by:

$$\lambda_{w;i,j,k,r} = \sum_{i'=1}^{I}\sum_{j'=1}^{J}\sum_{k'=1}^{K}\sum_{s'=1}^{S}\left(\overbrace{M_{w;i,j,k,i',j',k'}}^{\text{mixing matrix}}\overbrace{\frac{Y_{m;i',j',k',r',s'}}{X_{m;i',j',k'} + Y_{m;i',j',k',r',s'}}}^{\substack{\text{HIV prevalence}\\ \text{in groups contacted}}}\overbrace{\nu_{1;r'}\nu_{2;s'}\nu_{3;n}\beta}^{\substack{\text{transmission}\\ \text{probability}}}\right)$$

where $r = r'$; that is, upon infection the subject acquires the same drug-resistance status as that of the infecting partner. The parameter β is the baseline biological probability of HIV transmission per partnership, and the ν parameters are modifiers of this baseline probability to reflect specifics of the partnership: $\nu_{1;r'}$ is a modifier by drug resistance status, as drug-resistant HIV may pay an evolutionary "cost" of being less transmissible; $\nu_{2;s'}$ is a modifier by disease stage, whereby people with acute or advanced HIV have an

elevated probability of transmission and people who are virally suppressed on ART do not transmit at all; and $\nu_{3;n}$ is a modifier by partnership type (here, $n = 1$, heterosexual transmission to women from male partners; other possible values are $n = 2$ for heterosexual transmission to men from female partners and $n = 3$ for homosexual partnerships between MSM).

Model calibration

Historical data of HIV prevalence, incidence, and mortality were obtained from UNAIDS, with additional data stratified by age and sex obtained from ZIMPHIA and World Population Prospects. With carefully-chosen model inputs, our model outputs are within or close to the confidence intervals of historical data. We further fine-tuned our results by adjusting the phase-in period for ART. Comparison between model outputs and historical data are shown in **Figs. S4-S7**.

Development and computational environment

The model was developed and simulated using C++. Calculations and graphing of efficient frontiers were conducted using R. Probabilistic sensitivity analyses were set up and conducted using Python.

Tables and Figures

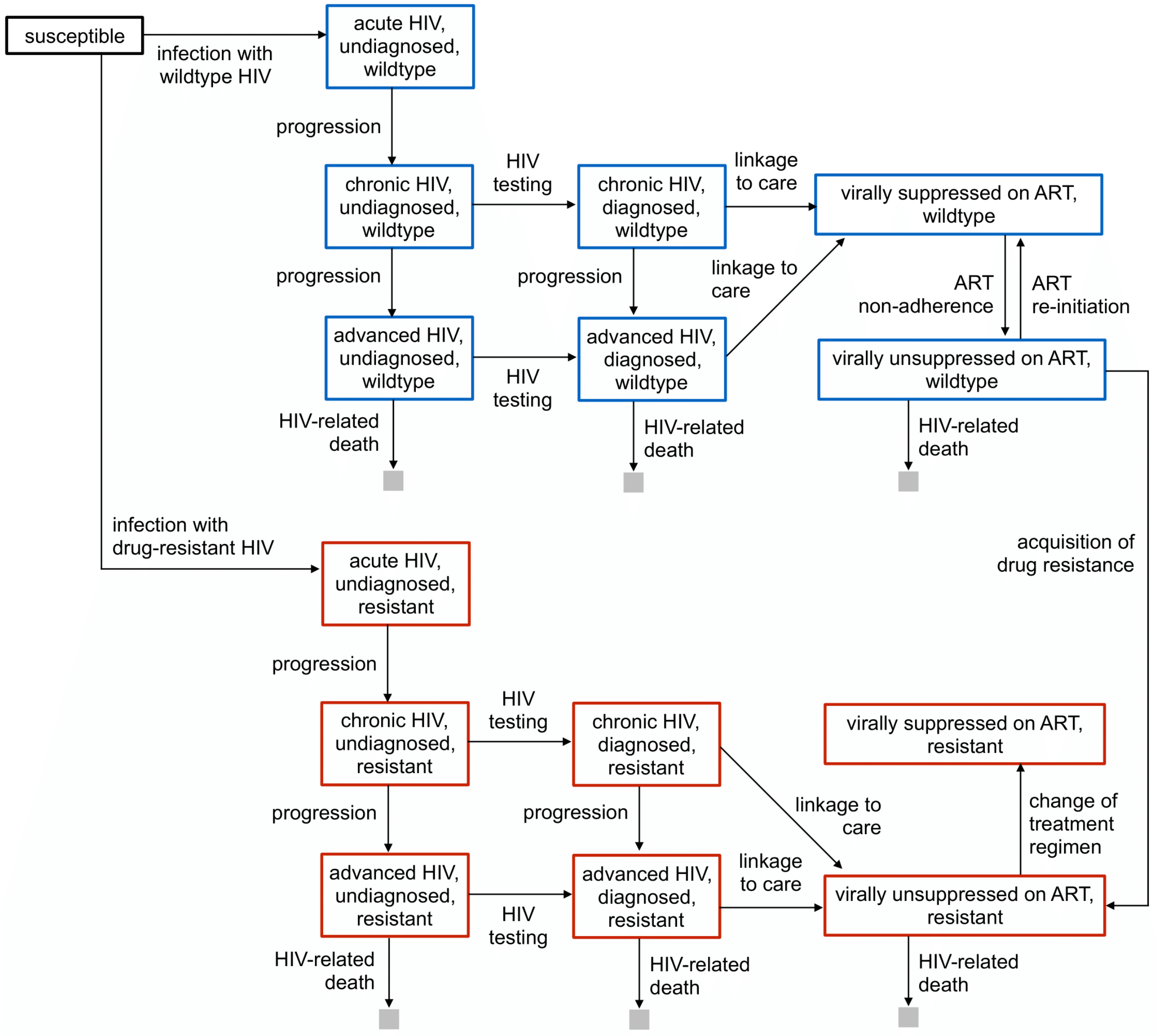

Fig. S1: Model structure for HIV progression and the care cascade.

Parameter	Value		Reference
Total Population			
Age			
0-14	38.3%		[1]
15-64	57.2%		[1, 2]
65+	4.5%		[1]
HIV risk behaviors	**Female**	**Male**	
Abstinent	36.3%	29.1%	[3-5]
Low risk (monogamous)	52.7%	44.0%	[3]
Moderate risk (multiple, concurrent relationships)	9.8%	23.2%	[4]
High risk (sex workers or clients of sex workers)	1.2%	3.7%	[5]
Alcohol Use Disorder[1]			
Prevalence			
WSM[2]	4.5%		[1, 2, 6-10][4]
MSW[2]	31.3%		[1, 2, 6-10][4]
MSM[2]	44.0%		[1, 2, 6-10][4]
Effect on			
Rate of partner change	+9.0%		[11]
HIV testing	-70.0%		[12]
Linkage to care	-65.0%		[13]
Adherence to ART	-39.0%		[14]
HIV transmission and risk behaviors			
Annual probability of diagnosis (if undiagnosed)	20.2%		[15]
Annual probability of linkage to care after a positive HIV test			
Chronic stage (CD4 ≥ 200)	88.9%		[16, 17]
Advanced stage (CD4 < 200)	59.3%		[16, 17]
Probability of adherence to ART (proportion of doses taken as directed)	63.0%		[18-22]
Annual probability of ART regimen change in response to resistance	33.0%		[23]
Probability of virologic suppression			
Perfect adherence	95%		[24]
Average adherence	60%		[18-22, 24][4]
Sexual risk behaviors			
Annual rate of partner change	**Female**	**Male**	
Low risk	0.02	0.13	[25, 26]
Moderate risk	1.21	3.32	[26]
High risk	6.06	6.65	[25, 26]
Assortativity[3] of sexual mixing by			
Partner change rate	0.32		[27]
Age	0.29		[27]
CASM	0.20		[28]

Fig. S2: Summary of essential model inputs

Outcomes impacted by interventions	Effects	Coverage (Current)	Coverage (Goal)	
HIV incidence rate				
Voluntary Medical Male Circumcision (VMMC) – 1 time	OR 0.44	23%	90%	[29-31]
Long-Acting PrEP (100% adherence)	HR 0.23	0%	70%	[32, 33]
Oral PrEP (30% adherence)	HR 0.74	10%	70%	[34, 35]
HIV diagnosis				
Community-based testing and counseling	RR 2.42	31%	90%	[36-38]
Index tracing	RR 1.48	57.5%	100%	[39], Administrative data, OPHID]
HIV Self-Testing	RR 1.42	0.5%	90%	[40, 41], [Administrative data, FHI360]
Linkage to care				
Defaulter tracing	OR 1.34	40%	81%	[42], [Administrative data, OPHID]
ART adherence				
Differentiated care	RR 1.43	22%	55%	[37, 43]

Fig. S3: Summary of intervention inputs

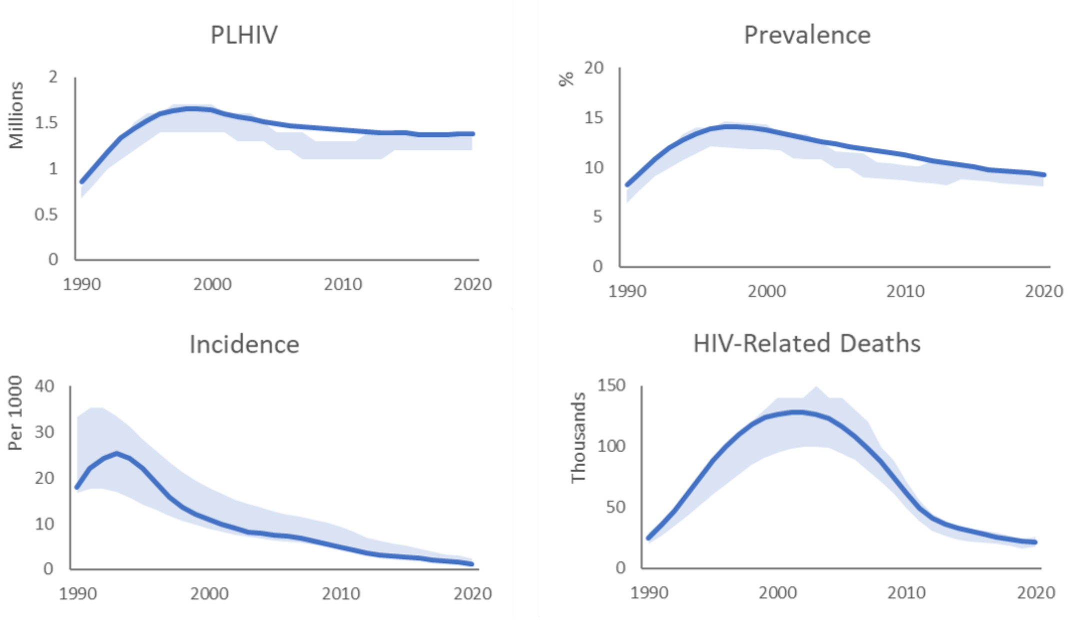

Fig. S4: Calibration results by total population: Model output (solid lines) vs. historical data (shaded region). Historical data are from UNAIDS.

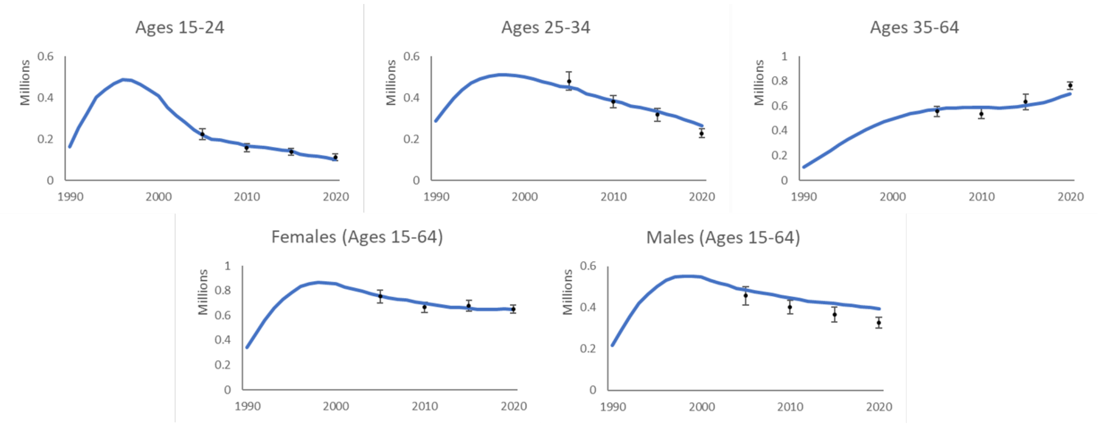

Fig. S5: PLHIV calibration results stratified by age and sex: Model output (solid lines) vs. data (vertical lines with point estimates). Point estimates are from ZIMPHIA and World Population Prospects.

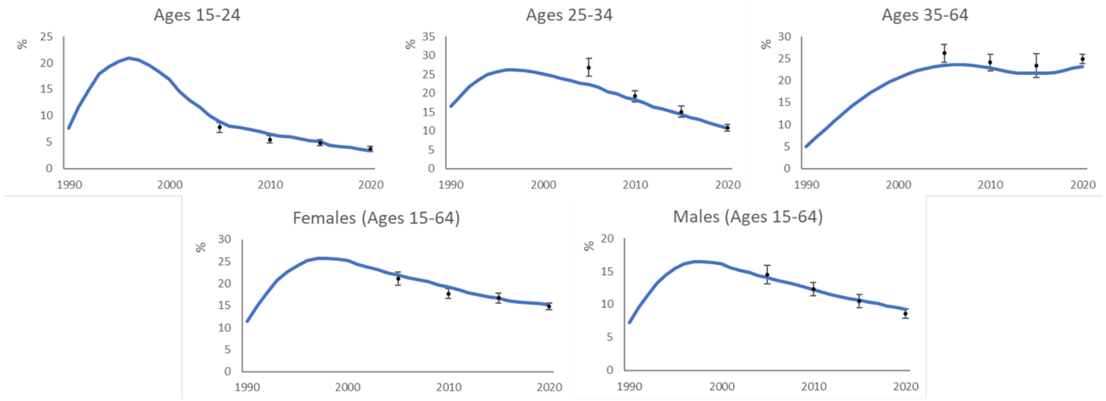

Fig. S6: Prevalence calibration results stratified by age and sex: Model output (solid lines) vs. data (vertical lines with point estimates). Point estimates are from ZIMPHIA and World Population Prospects.

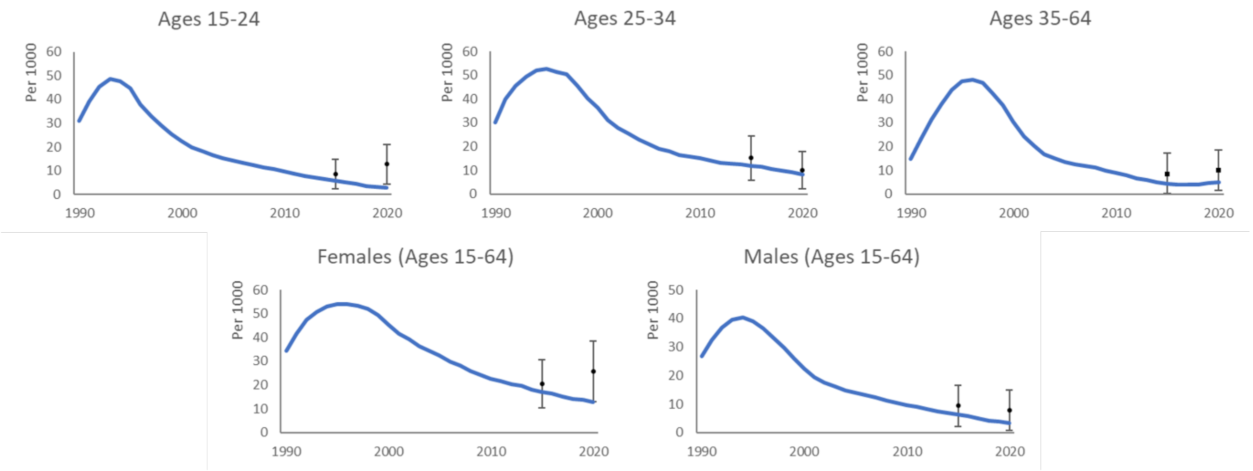

Fig. S7: Incidence calibration results stratified by age and sex: Model output (solid lines) vs. data (vertical lines with point estimates). Point estimates are from ZIMPHIA and World Population Prospects.

References

1. The World Factbook. Zimbabwe [Internet]; 2020 [cited 2024]. Available from: https://www.cia.gov/the-world-factbook/countries/zimbabwe/.

2. Index Mundi. Zimbabwe Age Structure [Internet]; 2020 [cited 2024]. Available from: https://www.indexmundi.com/zimbabwe/age_structure.html#google_vignette.

3. Chemaitelly H, Shelton JD, Hallett TB, Abu-Raddad LJ. Only a fraction of new HIV infections occur within identifiable stable discordant couples in sub-Saharan Africa. Aids. 2013;27(2):251-60.

4. Zimbabwe Demographic and Health Survey 2015: Final Report. Zimbabwe National Statistics Agency and ICF International: Rockville, Maryland, USA; 2016.

5. Fearon, E, Chabata ST, Magutshwa S, Ndori-Mharadze T, Musemburi S, Chidawanyika H, et al. Estimating the Population Size of Female Sex Workers in Zimbabwe: Comparison of Estimates Obtained Using Different Methods in Twenty Sites and Development of a National-Level Estimate. J Acquir Immune Defic Syndr 2020;85(1):30-38.

6. World Health Organization. Global status report on alcohol and health [Internet]; 2018 [cited 2024]. Available from: https://www.who.int/publications/i/item/9789241565639.

7. Grant BF, Goldstein RB, Saha TD, Chou SP, Jung J, Zhang H, Pickering RP, et al. Epidemiology of DSM-5 Alcohol Use Disorder: Results From the National Epidemiologic Survey on Alcohol and Related Conditions III. JAMA Psychiatry. 2015 Aug;72(8):757-66.

8. Bohm MK, Liu Y, Esser MB, Mesnick JB, Lu H, Pan Y, et al. Binge Drinking Among Adults, by Select Characteristics and State - United States, 2018. MMWR Morb Mortal Wkly Rep. 2021 Oct 15;70(41):1441-1446.

9. Vellios NG, Van Walbeek CP. Self-reported alcohol use and binge drinking in South Africa: Evidence from the National Income Dynamics Study, 2014 - 2015. S Afr Med J. 2017 Dec 13;108(1):33-39.

10. Pengpid S, Peltzer K. Prevalence and Correlates of Heavy Episodic Alcohol Consumption among Adults in Ecuador: Results of the First National STEPS Survey in 2018. Int J Environ Res Public Health. 2020 Dec 3;17(23):9017.

11. Belus JM, Baucom DH, Wechsberg WM. Individual and Relationship Predictors of Couple-Level Sexual Concurrency in Heterosexual South African Couples. Arch Sex Behav. 2020 Apr;49(3):999-1015.

12. Jooste S, Mabaso M, Taylor M, North A, Shean Y, Simbayi LC. Socio-economic differences in the uptake of HIV testing and associated factors in South Africa. BMC Public Health. 2021 Aug 26;21(1):1591.

13. Maughan-Brown B, Harrison A, Galárraga O, Kuo C, Smith P, Bekker LG, et al. Factors affecting linkage to HIV care and ART initiation following referral for ART by a mobile health clinic in South Africa: evidence from a multimethod study. J Behav Med. 2019 Oct;42(5):883-897.

14. Been SK, Schadé A, Bassant N, Kastelijns M, Pogány K, Verbon A. Anxiety, depression and treatment adherence among HIV-infected migrants. AIDS Care. 2019 Aug;31(8):979-987.

15. Zimbabwe Population-based HIV Impact Assessment (ZIMPHIA) 2015-2016: Final Report. Ministry of Health and Child Care (MOHCC), Zimbabwe: Harare, Zimbabwe; 2019.

16. Perelman J, Rosado R, Ferro A, Aguiar P. Linkage to HIV care and its determinants in the late HAART era: a systematic review and meta-analysis. AIDS Care. 2018 Jun;30(6):672-687.

17. Zetola NM, Bernstein K, Ahrens K, Marcus JL, Philip S, Nieri G, et al. Using surveillance data to monitor entry into care of newly diagnosed HIV-infected persons: San Francisco, 2006-2007. BMC Public Health. 2009 Jan 14;9:17.

18. Sithole Z, Mbizvo E, Chonzi P, Mungati M, Juru TP, Shambira G, et al. Virological failure among adolescents on ART, Harare City, 2017- a case-control study. BMC Infect Dis. 2018 Sep 18;18(1):469.

19. Erlwanger AS, Joseph J, Gotora T, Muzunze B, Orne-Gliemann J, Mukungunugwa S, et al. Patterns of HIV Care Clinic Attendance and Adherence to Antiretroviral Therapy Among Pregnant and Breast-feeding Women Living With HIV in the Context of Option B+ in Zimbabwe. J Acquir Immune Defic Syndr. 2017 Jun 1;75 Suppl 2:S198-S206.

20. Vreeman RC, Scanlon ML, Tu W, Slaven JE, McAteer CI, Kerr SJ, et al. Validation of a self-report adherence measurement tool among a multinational cohort of children living with HIV in Kenya, South Africa and Thailand. J Int AIDS Soc. 2019 May;22(5):e25304.

21. Haas AD, Msukwa MT, Egger M, Tenthani L, Tweya H, Jahn A, et al. Adherence to Antiretroviral Therapy During and After Pregnancy: Cohort Study on Women Receiving Care in Malawi's Option B+ Program. Clin Infect Dis. 2016 Nov 1;63(9):1227-1235.

22. Davies MA, Boulle A, Fakir T, Nuttall J, Eley B. Adherence to antiretroviral therapy in young children in Cape Town, South Africa, measured by medication return and caregiver self-report: a prospective cohort study. BMC Pediatr. 2008 Sep 4;8:34.

23. Chimbetete C, Shamu T, Keiser O. Zimbabwe's national third-line antiretroviral therapy program: Cohort description and treatment outcomes. PLoS One. 2020 Mar 2;15(3):e0228601.

24. Byrd KK, Hou JG, Hazen R, Kirkham H, Suzuki S, Clay PG, et al. Antiretroviral Adherence Level Necessary for HIV Viral Suppression Using Real-World Data. J Acquir Immune Defic Syndr. 2019 Nov 1;82(3):245-251.

25. Nsubuga RN, White RG, Mayanja BN, Shafer LA. Estimation of the HIV basic reproduction number in rural south west Uganda: 1991-2008. PLoS One. 2014 Jan 3;9(1):e83778.

26. Gregson S, Mugurungi O, Eaton J, Takaruza A, Rhead R, Maswera R, et al. Documenting and explaining the HIV decline in east Zimbabwe: the Manicaland General Population Cohort. BMJ Open. 2017 Oct 6;7(10):e015898.

27. Malagón T, Burchell A, El-Zein M, Tellier PP, Coutlée F, Franco EL; HITCH study group. Assortativity and Mixing by Sexual Behaviors and Sociodemographic Characteristics in Young Adult Heterosexual Dating Partnerships. Sex Transm Dis. 2017 Jun;44(6):329-337.

28. Prah P, Copas AJ, Mercer CH, Nardone A, Johnson AM. Patterns of sexual mixing with respect to social, health and sexual characteristics among heterosexual couples in England: analyses of probability sample survey data. Epidemiol Infect. 2015 May;143(7):1500-10.

29. Sharma SC, Raison N, Khan S, Shabbir M, Dasgupta P, Ahmed K. Male circumcision for the prevention of human immunodeficiency virus (HIV) acquisition: a meta-analysis. BJU Int. 2018 Apr;121(4):515-526.

30. Zimbabwe Population-based HIV Impact Assessment 2020 (ZIMPHIA 2020): Final Report. Ministry of Health and Child Care (MoHCC): Harare, Zimbabwe; 2021.

31. World Health Organization. A framework for voluntary medical male circumcision: effective HIV prevention and a gateway to improved adolescent boys' & men's health in Eastern and Southern Africa by 2021 [Internet]; 2016 [cited 2024]. Available from: https://www.who.int/publications/i/item/WHO-HIV-2016.17.

32. Landovitz RJ, Donnell D, Clement ME, Hanscom B, Cottle L, Coelho L, et al; HPTN 083 Study Team. Cabotegravir for HIV Prevention in Cisgender Men and Transgender Women. N Engl J Med. 2021 Aug 12;385(7):595-608.

33. HIV Prevention Trials Network. HPTN 084 Study Demonstrates Superiority of CAB LA to Oral

TDF/FTC for the Prevention of HIV [Internet]; 2020 [cited 2024]. Available from: https://www.hptn.org/news-and-events/press-releases/hptn-084-study-demonstrates-superiority-of-cab-la-to-oral-tdfftc-for.

34. McCormack S, Dunn DT, Desai M, Dolling DI, Gafos M, Gilson R, et al. Pre-exposure prophylaxis to prevent the acquisition of HIV-1 infection (PROUD): effectiveness results from the pilot phase of a pragmatic open-label randomised trial. Lancet. 2016 Jan 2;387(10013):53-60.

35. Ncube, G., National PrEP Program Update (January - April 2022). MOHCC AIDS and TB Programme: Zimbabwe; 2022.

36. Sharma M, Ying R, Tarr G, Barnabas R. Systematic review and meta-analysis of community and facility-based HIV testing to address linkage to care gaps in sub-Saharan Africa. Nature. 2015 Dec 3;528(7580):S77-85.

37. Apollo, T. Update of Zimbabwe context: successes & challenges, Allocative Efficiency Modelling Stakeholders' Workshop. Ministry of Health and Child Care (MOHCC); 2022.

38. Suthar AB, Ford N, Bachanas PJ, Wong VJ, Rajan JS, Saltzman AK, et al. Towards universal voluntary HIV testing and counselling: a systematic review and meta-analysis of community-based approaches. PLoS Med. 2013 Aug;10(8):e1001496.

39. Cherutich P, Golden MR, Wamuti B, Richardson BA, Ásbjörnsdóttir KH, Otieno FA, et al; aPS Study Group. Assisted partner services for HIV in Kenya: a cluster randomised controlled trial. Lancet HIV. 2017 Feb;4(2):e74-e82.

40. Johnson CC, Kennedy C, Fonner V, Siegfried N, Figueroa C, Dalal S, et al. Examining the effects of HIV self-testing compared to standard HIV testing services: a systematic review and meta-analysis. J Int AIDS Soc. 2017 May 15;20(1):21594.

41. Muchedzi A, Mpofu M, Mudzengerere FH, Bateganya M, Mavimba T, Satti H, et al. High HIV Positivity Rates Following Large-Scale HIV Self-Testing Implementation in Zimbabwe, 2018-2020. Front Public Health. 2021 Mar 23;9:606376.

42. Bershetyn A, Odeny TA, Lyamuya R, Nakiwogga-Muwanga A, Diero L, Bwana M, et al; East Africa International Epidemiologic Databases to Evaluate AIDS (EA-IeDEA) Consortium. The Causal Effect of Tracing by Peer Health Workers on Return to Clinic Among Patients Who Were Lost to Follow-up From Antiretroviral Therapy in Eastern Africa: A "Natural Experiment" Arising From Surveillance of

Lost Patients. Clin Infect Dis. 2017 Jun 1;64(11):1547-1554.

43. Eshun-Wilson I, Awotiwon AA, Germann A, Amankwaa SA, Ford N, Schwartz S, et al. Effects of community-based antiretroviral therapy initiation models on HIV treatment outcomes: A systematic review and meta-analysis. PLoS Med. 2021 May 28;18(5):e1003646.
